# Efficacy of a video‐viewing intervention of smartphone‐based positive word stimulation on depressive symptoms in individuals with subthreshold depression: Protocol for a randomized controlled trial

**DOI:** 10.1002/pcn5.70284

**Published:** 2026-01-31

**Authors:** Hiroyuki Uchida, Takumi Igusa, Chihaya Machida, Tomohiro Shimada, Kazuki Hirao

**Affiliations:** ^1^ Graduate School of Health Sciences Gunma University Maebashi Japan; ^2^ Department of Rehabilitation Kurashiki Heisei Hospital Kurashiki Japan; ^3^ Department of Rehabilitation Fujioka General Hospital Fujioka Japan; ^4^ Department of Rehabilitation, Medical Corporation Taiseikai Uchida Hospital Numata Japan; ^5^ Department of Occupational Therapy, School of Health Sciences, Faculty of Medicine Kagoshima University Kagoshima Japan

**Keywords:** depression, mental health, MHealth, smartphone, subthreshold depression

## Abstract

**Background:**

Interventions targeting subthreshold depression (StD) are critically important for preventing major depressive disorder's onset. Our smartphone‐based, video‐viewing website (SPSRS), which presents positive word stimulation in videos, is a novel intervention for improving StD's depressive symptoms. Because no rigorous evidence exists for SPSRS's efficacy, this trial aims to evaluate the SPSRS intervention's efficacy on depressive symptoms.

**Methods:**

The trial will be an evaluator‐blinded, randomized, 5‐week, parallel‐group superiority trial designed to assess whether the SPSRS intervention (experimental group) is effective compared to no intervention (waiting‐list control group). In a 1:1 ratio, 400 participants with StD will be randomly assigned to an experimental group (*n* = 200) or a waiting‐list control group (*n* = 200). The experimental group will receive a 10‐min SPSRS video‐viewing intervention once daily for 5 weeks; when the intervention ends, the waiting‐list control group will receive the treatment upon request. The intervention's primary outcome will be the mean change from baseline on the Center for Epidemiologic Studies Depression Scale; secondary outcomes will include the mean change from baseline on the Kessler Psychological Distress Scale and the Generalized Anxiety Disorder seven‐item scale.

**Result:**

The results are presented according to the protocol.

**Discussion:**

This trial's confirmation of SPSRS's efficacy could lead to new StD intervention strategies and provide interventions tailored to patient needs.

## INTRODUCTION

Over the past few decades, the clinical significance of subthreshold depression (StD) (e.g., subsyndromal, subclinical, or minor depression) has received considerable attention.^1^, ^2^ StD does not meet diagnostic criteria for major depressive disorder (MDD) but is considered present when an individual experiences clinically significant depressive symptoms measured by patient‐reported outcomes (PROs).^3^ StD occurs across all age groups, and a systematic review indicated its prevalence at 11.02%.^2^, ^4^ Moreover, StD causes such problems as diminished daily functioning, reduced quality of life, worsening health status, increased economic costs, and increased mortality.^3^, ^5^, ^6^, ^7^, ^8^, ^9^, ^10^ But particularly important is that studies have indicated that StD exists on the spectrum of MDD and that individuals with StD are more likely to transition to MDD.^1^, ^2^, ^6^ Therefore, StD interventions' development and implementation are essential not only for improving various problems caused by StD but also for preventing the onset of MDD, a major public health issue.

Systematic reviews have demonstrated that psychotherapy improves depressive symptoms in individuals with StD and thus may reduce the risk of MDD onset.^11^, ^12^ Therefore, investigating psychotherapy's therapeutic efficacy further and promoting its usage is important. However, developing and investigating various other treatment options is necessary because studies have suggested that considering patients' treatment preferences and approaches is necessary to motivate treatment adherence, prevent treatment dropout, and lead to more efficacious mental‐health outcomes.^13^, ^14^, ^15^ Given patient preferences' influence on treatment's efficacy, developing a variety of treatment strategies to reduce StD's depressive symptoms is crucial.

Our newly developed, smartphone‐based video‐viewing website (SPSRS) can serve as such an intervention strategy.^16^ Designed based on word stimulation research and positive words from a qualitative study of patients with StD, SPSRS displays two words of stimulation to improve depressive symptoms and prevent progression to MDD.^17^, ^18^, ^19^, ^20^ The SPSRS website has the advantage of free use on ever‐more‐ubiquitous smartphones, thereby reaching many who can benefit from treatment.^21^, ^22^, ^23^ Significantly, SPSRS uses the YouTube application programming interface (API), enabling users to watch videos uploaded to YouTube directly via SPSRS. Thus, people with StD can choose preferred, relevant videos and have fun working toward improving their depressive symptoms.

To date, several studies have demonstrated SPSRS's efficacy.^24^, ^25^, ^26^, ^27^, ^28^, ^29^ First, a study investigating its feasibility found it safe and easy to use, with no adverse events (AEs) during intervention.^16^ After a 5‐week intervention, a pilot randomized controlled trial (RCT) showed moderate improvement in the experimental group's depressive symptoms (measured by the Center for Epidemiologic Studies Depression Scale [CES‐D, Japanese version]) compared to those of the waiting‐list control group.^25^, ^27^ This pilot's unique strength lies in its ability to improve StD depressive symptoms using CES‐D scores.

Of various PROs identifying individuals with StD and assessing changes in depressive symptoms, the widely used CES‐D effectively evaluates StD.^2^, ^30^ Importantly, the CES‐D score offers advantages over other PROs (e.g., the Beck Depression Inventory‐II [BDI‐II], the Patient Health Questionnaire‐9 [PHQ‐9]) when evaluating SPSRS's intervention efficacy on StD depressive symptoms. The CES‐D score includes four factors: (1) somatic and retarded activity, (2) depressed affect, (3) positive affect, and (4) interpersonal problems.^31^ Specifically, when measuring depressive symptoms, other PROs (e.g., BDI‐II, PHQ‐9) do not include positive affect, but the CES‐D's total score indicates symptoms' severity based on whether a person has experienced a depressive episode and whether they feel happy, making it possible to measure depressive symptoms' various aspects in detail.^31^, ^32^, ^33^ Especially because SPSRS uses positive words to improve depressive symptoms, the CES‐D containing positive items is likely the most suitable PRO for accurately measuring SPSRS's intervention efficacy. Therefore, the previously conducted pilot RCT appropriately evaluated SPSRS's efficacy with the patients with StD population.

Notably, no formal sample size calculation was performed for the previous pilot RCT; however, it was conducted with the minimum sample size to ensure the results' scientific validity (16 participants per group, 32 participants total).^25^ Even so, data from small samples inherently contain inaccuracies.^34^, ^35^, ^36^ Therefore, to determine SPSRS's intervention efficacy, the pilot's changes in CES‐D scores are preliminary, emphasizing the need for a full‐scale RCT based on a formal sample size.

### Primary hypothesis and objectives

To that end, we have planned an RCT with the primary objective of verifying SPSRS's efficacy for individuals with StD. We hypothesize that the SPSRS intervention (experimental group) will be effective in reducing depressive symptoms, as assessed by the CES‐D, in individuals with StD compared to no intervention (waiting‐list control group).

### Identifying optimal intervention timing to enhance SPSRS intervention's efficacy

If SPSRS proves efficacious in reducing StD's depressive symptoms, estimating its appropriate intervention duration is crucial, especially because its video‐viewing time has not yet been sufficiently established. Identification of appropriate intervention duration not only enables theoretical refinement of the therapeutic mechanism and SPSRS process but can also help clarify its clinical implementation. Therefore, the trial's secondary objective is to explore an appropriate intervention duration.

## METHODS AND ANALYSIS

### Trial design

This trial is a 5‐week, evaluator‐blinded, 1:1 parallel‐group superiority RCT to determine SPSRS efficacy. It was designed in accordance with the Recommendations for Interventional Trials (SPIRIT) 2025 statement and SPIRIT‐PRO.^37^, ^38^

### Eligibility

Participants will be selected according to the following.

#### Inclusion criteria

(1) Male or female.
(2) 18–64 years old.
(3) CES‐D score of 16 or higher.^31^, ^39^
(4) Own a smartphone.
(5) Have provided electronic informed consent before participation.

#### Exclusion criteria

(1) Individuals with a lifetime history of mental illness.
(2) Individuals receiving treatment from mental‐health professionals.
(3) Persons with visual or hearing impairments that adversely affect their daily lives.
(4) PHQ‐9 Item 1 (Little interest or pleasure in doing things) or Item 2 (Feeling down, depressed, or hopeless) with responses of “several days,” “more than half the days,” or “nearly every day,” and who also responded “more than half the days” or “nearly every day” to five of nine items.^39^

Generally, StD does not meet diagnostic criteria for MDD but is considered present when an individual experiences clinically significant depressive symptoms as measured by PRO.^3^ However, because a market research company will conduct this trial, ruling out MDD based on structured interviews such as the Mini‐International Neuropsychiatric Interview (MIINI) will be difficult.^40^ In such cases, one approach is to use the PRO Depression Scale to rule out MDD.^39^, ^41^, ^42^, ^43^, ^44^, ^45^ In a previous study, individuals were judged to have MDD if, in addition to having a CES‐D score of 16 or higher, they responded “several days,” “more than half the days,” or “nearly every day” to PHQ‐9 Item 1 (Little interest or pleasure in doing things) or Item 2 (Feeling down, depressed, or hopeless), and responded “more than half the days” or “nearly every day” to five or more of the nine items.^39^ In contrast, those with a CES‐D score of 16 or higher who did not meet the PHQ‐9 criteria for MDD were judged to have StD.^39^ In this trial, StD is defined according to the identification method used in the previous study.

### Study setting and recruitment timeline

**Figure 1:**
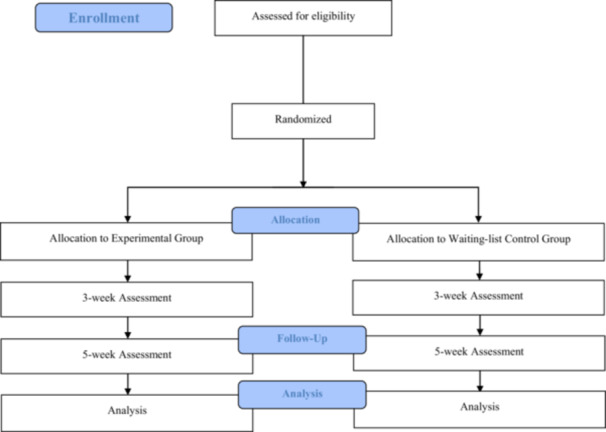
CONSORT flowchart of study design.

This trial will recruit participants from the panel network (36 million members) of a Japanese market research company (Macromill Group, Tokyo) from October 1, 2025, to March 31, 2030. The research firm will email invitations to panelists aged 18–64, explaining the trial's purpose and procedures. The invitation will include an electronic consent option; individuals who check “consent” will be deemed to have agreed to participate in the main trial and can proceed to the online questionnaire for eligibility assessment. Participants who meet eligibility criteria will then undergo a baseline assessment and be considered enrolled. By the Central Registration Center at Kagoshima University, they will subsequently be randomly assigned to the experimental or the waiting‐list control group. Participants will be scheduled to complete Japanese versions of the CES‐D, Kessler Screening Scale for Psychological Distress (K‐6), and Generalized Anxiety Disorder‐7 (GAD‐7) questionnaires at 3 and 5 weeks after the intervention begins.^31^, ^46^, ^47^, ^48^, ^49^ Additionally, at 5 weeks after the intervention begins, the Global Rating of Change (GRC) scales will be assessed in addition to the CES‐D, K6, and GAD‐7.^50^, ^51^ The CES‐D will be administered during the main trial's eligibility assessment, while the K6 and GAD‐7 will be administered during baseline assessment. At 3 weeks after the intervention begins, assessments will be conducted in the following order: CES‐D, K6, and GAD‐7. At 5 weeks, assessments will be conducted in the following order: CES‐D, K6, GAD‐7, and GRC scales. Demographic information and lifestyle characteristics will be assessed only at baseline. Figure 1 shows the study's flowchart, and Table 1 shows the assessment schedule.

**Table 1:** Assessment schedule.

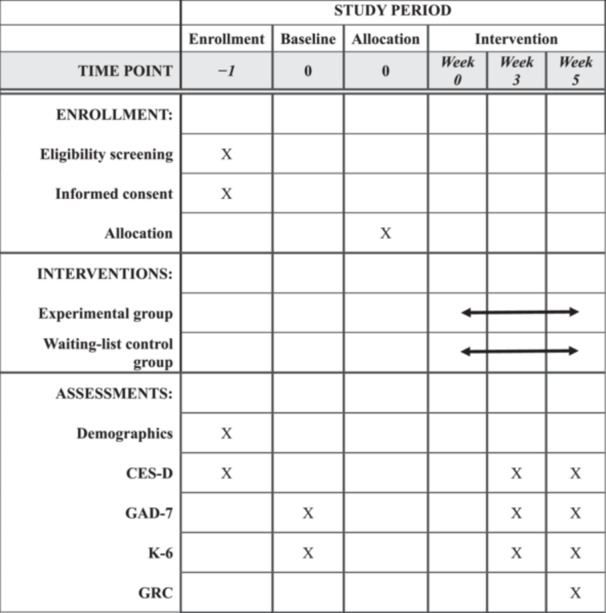

Abbreviations: CES‐D, center for epidemiologic studies depression scale; GAD‐7, generalized anxiety disorder‐7; GRC, global rating of change; K‐6, kessler screening scale for psychological distress.

### Interventions

#### Experimental group

The experimental group will receive a video‐viewing intervention using SPSRS, which allows participants to search for videos by keyword and watch them (similar to YouTube).^16^ To enable participants to access YouTube videos, SPSRS utilizes the YouTube API. Thus, to leverage SPSRS's benefits, participants will choose which videos to watch and enjoy.

The key difference between SPSRS and video‐viewing sites like YouTube is that SPSRS presents two types of word stimulation during the video. First, a confidence‐boosting word or phrase randomly appears (17 ms) in one of the screen's four corners: *can*, *let us try*, *good luck*, *able*, or *do not worry*.^17^ Immediately afterward, a positive word appears (150 ms) at the screen's center: *nice*, *great*, *fantastic*, *satisfactory*, or *enjoyable*.^18^ These words repeat every 5 s. The experimental group will engage in SPSRS video‐viewing for at least 10 min per day (at least 70 min per week; at least 350 min over 5 weeks), determined based on the previous pilot RCT.^25^

This trial will undoubtedly enroll participants with diverse preferences in video content, and each individual is free to select videos that interest them so that the intervention is applicable to a wide adult demographic. However, as the SPSRS utilizes videos from YouTube, participants will not have access to videos that violate YouTube's Community Guidelines (e.g., due to violent, sexual, or fraudulent content). Additionally, participants may pause or stop the video at any time if they experience discomfort or health effects while watching. While the SPSRS system does not currently have its own reporting function, users can directly navigate from the SPSRS video playback screen to YouTube and report violations.

Participants will receive the SPSRS link and instructions for its use via email. To increase adherence, a templated text reminder will be sent once a week.^25^, ^52^ Additionally, SPSRS not only displays the daily required viewing time on the home screen but also allows participants to check their current viewing time via the history function.

#### Waiting‐list control group

The waiting‐list control group will not receive instructions to implement the video‐viewing intervention using SPSRS or other websites (such as YouTube) during the 5‐week intervention period. However, if participants wish to use the SPSRS, they will receive the video‐viewing intervention using the SPSRS after completing a questionnaire 5 weeks after the intervention begins.

#### Relevant concomitant care and interventions permitted/prohibited during the trial

From an ethical standpoint, no restrictions on interventions will be imposed in this trial.

#### Criteria for discontinuing or modifying allocated interventions

(1) A participant wishes to discontinue the intervention, and continuing the intervention is determined to be difficult.
(2) The entire clinical trial is discontinued.
(3) A participant becomes ineligible after enrollment.
(4) The research team determines that discontinuing the intervention is warranted.

Participants are not considered to have withdrawn from the trial when the intervention ends and are requested to undergo primary and secondary outcome assessments. Participants who refuse to undergo assessment or withdraw consent are considered to have withdrawn. Participants will be excluded from the intention‐to‐treat (ITT) analysis only if they are deemed unable to meet eligibility criteria.

### Outcomes

This trial's primary outcome will be the mean change in the CES‐D's total score from baseline to the intervention's scheduled end (5 weeks).^31^ The secondary outcomes are mean changes in total scores of the K6 and the GAD‐7 from baseline to the intervention's end (5 weeks).^46^, ^47^, ^48^, ^49^

### Allocation

Following baseline assessment, participants will be randomly assigned to the experimental group or the waiting‐list control group in a 1:1 ratio. The randomization list will be generated by an independent third party using the blockrand package in R (version 4.5.1) with a replacement block method of appropriate size. To ensure allocation concealment, the block size will not be disclosed until the trial ends. The generated randomization list will be managed at the Central Registration Center at Kagoshima University, and participants will be randomly assigned after completing the baseline questionnaire. These procedures will prevent selection bias.

### Blinding

Due to the intervention's nature, blinding of participants and therapists is not possible, so only evaluators will be blinded. Baseline assessments will be performed before randomization. Additionally, to maintain evaluators' blinding, assessments at 3 and 5 weeks after the intervention begins will be automatically sent to participants via a templated online questionnaire provided by the market research company. Because the trial blinds only evaluators, the need for emergency unblinding is expected to be extremely low.

### Data collection

Table 1 shows the outcome assessments' schedule. Information regarding depressive symptoms (primary outcome), psychological distress, and anxiety symptoms will be collected at baseline, 3 and 5 weeks after the intervention begins. Additionally, as a baseline assessment, demographic data will be collected via online questionnaires: gender and age, lifestyle characteristics including sleep duration and exercise, drinking, and smoking habits. To protect users' privacy, the research team will not track the specific video content viewed by participants. Instead, only participants' video‐viewing time will be collected using the SPSRS video‐viewing time recording site managed by the principal investigator. To prevent missing data, the questionnaires are set to reject incomplete responses automatically.

#### Patient Health Questionnaire‐9

The PHQ‐9 (Japanese version) is a 9‐item self‐report questionnaire designed to measure depressive symptoms.^53^ Each item is rated on a scale of 0–3 (0 = not at all, 1 = several days, 2 = more than half the days, and 3 = nearly every day), with a total score from 0 to 24. Higher total scores indicate greater depressive symptoms. Previous studies have validated the PHQ‐9's reliability and validity.^53^, ^54^, ^55^, ^56^, ^57^, ^58^, ^59^, ^60^ Additionally, the PHQ‐9 identifies the presence of MDD.^39^

#### Center for Epidemiologic Studies Depression Scale

The CES‐D (Japanese version) is a 20‐item self‐report questionnaire that measures depressive symptoms on four subscales: (1) somatic and retarded activity; (2) depressed affect; (3) positive affect; and (4) interpersonal problems.^31^ The CES‐D uses a four‐point Likert scale (A ≤ 1 day, B = 1–2 days, C = 3–4 days, and D = 5–7 days), with each item scored from 0 to 3. Total scores range from 0 to 60, with higher scores indicating more severe depressive symptoms. The CES‐D's reliability and validity have been verified.^31^, ^61^, ^62^, ^63^, ^64^

#### Kessler Screening Scale for Psychological Distress

The K‐6 (Japanese version) is a six‐item self‐report questionnaire designed to measure psychological distress.^47^ Each item is rated on a scale of 0–4 (4 = all of the time, 3 = most of the time, 2 = some of the time, 1 = a little of the time, and 0 = none of the time), with a total score from 0 to 24. Higher scores indicate greater psychological distress. Previous studies have confirmed the K‐6's reliability and validity.^47^, ^48^, ^62^, ^63^, ^65^

#### Generalized Anxiety Disorder 7‐item Scale

The GAD‐7 (Japanese version) is a 7‐item self‐report questionnaire designed to measure generalized anxiety disorder.^49^ Each item is rated on a scale of 0 to 3 (0 = not at all sure, 1 = several days, 2 = over half the days, and 3 = nearly every day). The total score ranges from 0 to 21, with higher scores indicating greater anxiety symptoms. Previous studies have investigated the GAD‐7's reliability and validity.^49^, ^54^, ^62^, ^66^, ^67^, ^68^

#### Global Rating of Change Scale

The GRC scales (Japanese version) is a single self‐report measure for assessing subjective well‐being over time.^50^, ^51^ Participants select the most appropriate response from five options (1 = I feel a lot better, 2 = I feel slightly better, 3 = I feel about the same, 4 = I feel slightly worse, and 5 = I feel a lot worse) to the question: “Compared to when we last saw you 5 weeks ago, how have your moods and feelings changed?”

### Plans to promote participant retention and complete follow‐up

To increase participant retention rates, several strategies will be employed. Participants will be informed that in addition to the use of SPSRS, baseline, 3‐week, and 5‐week post‐intervention outcome measurements will be provided free of charge. Besides that, participants can freely select videos to watch. Because waiting‐list control group participants might wish to receive the video‐viewing intervention, they will be informed that they can receive it when the formal trial ends after 5 weeks. All participants who complete the outcome measurements at 3 and 5 weeks after the intervention begins will receive a total of 200 redeemable points (100 at each measurement point) from the market research company that may be used to obtain gift cards or cryptocurrency.^69^ These incentives compensate participants for the time spent responding to each survey and are provided to maximize data collection for verifying intervention effects. Furthermore, these incentives will serve to mitigate risks such as gaming fund diversion and reward misuse.^69^ In addition, since other expenses such as data charges and electricity bills are expected to be higher in the intervention group than the waiting‐list control group, participants in the intervention group will receive an additional 2300 points if they respond to scheduled outcome measurements 3 and 5 weeks after the intervention begins based on a contract with the market research company.

All outcome assessments will be conducted via online surveys to accommodate participants' schedules. To encourage continued participation at each survey point (3 and 5 weeks after the intervention begins), reminders will be sent via email through the market research company to participants who have not completed the survey. Reminders will be sent once per day for a maximum of 2 days following the initial request.

Online surveys are configured to prevent incomplete responses, thereby addressing data loss. Participants who meet criteria for intervention refusal or discontinuation will be asked to provide data and participate in outcome assessments at 3 and 5 weeks after intervention initiation. These explanations and requests will be emphasized and explained during the electronic informed consent process.

### Sample size

In the previous 5‐week pilot RCT, Hedge's g for CES‐D scores between the SPSRS and control groups was 0.61.^25^ However, pilot studies have been shown to overestimate results.^34^, ^35^, ^36^ Therefore, considering a Hedge's *g* of 0.4, a two‐sided significance level of 5%, and a power of 80%, the sample size was calculated at 100 participants per group, totaling 200. Furthermore, internet‐based studies have reported dropout rates of 30%, and a systematic review of RCTs in smartphone apps for depression indicated a dropout rate of 47.8% after adjustment for publication bias.^70^, ^71^ Therefore, assuming a 50% dropout rate, this trial's sample size, using the pwr package in R (version 4.5.1), was calculated at 200 participants per group, totaling 400.

### Statistical analysis

The latest version of R will be used for data analysis.

#### Analysis of primary and secondary outcomes

Data analysis will be based on ITT analysis. Therefore, participants will be analyzed based on their initial group assignment regardless of adherence to the intervention. Assuming primary outcome data are randomly missing, the trial will use a linear mixed model (LMM) with restricted maximum‐likelihood estimation to compare mean changes between the experimental and waiting‐list control groups from baseline to Week 5.^72^ LMM can address defects arising in clinical trials and use all available data to estimate results, thereby making it possible to compare the experimental group that addressed the missing data to the waiting‐list control group.^72^

The CES‐D score is the dependent variable; the independent variables are group (experimental or control), time (baseline, 3 weeks, 5 weeks), and interaction between group and time—all treated as fixed‐effect factors, with participants treated as random‐effect factors. Baseline CES‐D scores will be included as covariates in the LMM. Additionally, to confirm results' robustness for the primary outcome, sensitivity analyses will be conducted using multiple imputation methods and per‐protocol analyses only for participants who provided complete data.^73^ Statistical significance will be set at *p* < 0.05 using a two‐tailed test. Additionally, Hedge's *g* will be calculated as the effect size between the two groups.^74^, ^75^ Secondary outcomes will be analyzed using the same procedures as primary outcomes; however, due to secondary outcomes' exploratory nature, no adjustments for multiple comparisons will be performed. No subgroup analyses are currently planned. LMM calculations will use the lmerTest package, and Hedge's *g*‐statistic will be calculated using the compute.es package. Multiple comparisons will be performed using the mice package.

#### Estimating appropriate intervention time for SPSRS

The estimation of the appropriate SPSRS intervention time will use data from the experimental group. Each participant's reliable change (RC) in depressive symptoms will be defined as a change in the CES‐D score (baseline to 5 weeks) that exceeds the minimal important change (MIC). The appropriate SPSRS video‐viewing time will be estimated by the receiver operator characteristic (ROC) method, which calculates the area under the curve, sensitivity, specificity, and the Youden index.^76^ The maximum Youden index point will be the optimal SPSRS intervention time. A previous study has estimated the CES‐D score's MIC; however, the MIC may vary depending on the disease, the intervention, the follow‐up period, and the estimation method.^77^, ^78^, ^79^ Therefore, the CES‐D score's MIC used as this trial's RC will be estimated using a predictive modeling method anchored to the GRC scales after the experimental group's 5‐week intervention.^78^, ^79^ The ROC method will be implemented with the pROC package, and the predictive modeling method will be conducted with the stats package.

### Data management, access to data, and confidentiality

Data entry and statistical analysis will be performed using Kurashiki Heisei Hospital computers unconnected to the Internet. Data from market research companies can be downloaded by logging on to the company websites. An ID and password are required to enable login, which will be sent only to the research manager by the market research company. Furthermore, data downloads are available for 30 days, after which the data will be deleted and downloads no longer possible. Survey data are provided in an anonymized state with participant numbers assigned. Additionally, no information regarding participants' identities will be provided or entered into an electronic database. All participant data will be entered into an Excel database stored on a USB drive equipped with password protection and encryption (AES‐256) and stored in a lockable cabinet at Kurashiki Heisei Hospital to maintain confidentiality. Additionally, USB memory passwords will be managed in a separate locked cabinet to prevent incidents such as information leaks or loss. In the event of a data breach or loss, the content of the breach and its potential impact shall be promptly investigated. The findings shall be reported to the Ethics Review Committee of Kurashiki Heisei Hospital, which will determine the appropriate response. Cross‐sectional data will be periodically sent from the survey company to Kurashiki Heisei Hospital. After the research team's error checking, these data will be cumulatively added to the previous data.

To ensure the entered data's accuracy, three researchers will conduct a two‐step verification. First, two researchers will independently enter data into Excel spreadsheets. To verify consistency, the third researcher will then compare the data sent by the research company with the two previously entered datasets. Only the data manager and principal investigator have database access, and they will complete the statistical‐analysis dataset. Changes and updates to these databases will be recorded and monitored using Windows Event Viewer and Excel history functions. In accordance with the principles of role‐based access control, only data managers and principal investigators possess database access rights, and they will finalize the datasets for statistical analysis. At this time, there are no plans to release the datasets to third parties. Furthermore, no secondary analyses are currently scheduled. However, if access to the data is requested for purposes such as secondary analysis or meta‐analysis, it will be granted only after obtaining renewed approval from the ethics review committee (in addition to any third‐party ethics committee) and renewed informed consent from the participants. All this study's obtained information will be stored at Kurashiki Heisei Hospital for 10 years.

### Data monitoring

Video‐based interventions are non‐invasive and are not expected to cause serious harm. In fact, there have been no reports of AEs resulting from the use of SPSRS by individuals with StD. Furthermore, since data collection will be conducted using an online survey, there are currently no plans to establish a formal data monitoring committee or to conduct an interim analysis of intervention efficacy or trial monitoring.

### Adverse events and post‐trial care

Multiple studies have examined SPSRS as a treatment for StD using the same 5‐week intervention period, and none have reported serious AEs, such as the onset of MDD.^16^, ^24^, ^25^, ^29^ Nonetheless, the possibility of such adverse effects on participants' physical and mental health should never be discounted during clinical trials. Therefore, we will employ methods similar to those used in previous studies to systematically monitor the occurrence and signs of AEs and respond promptly.^24^, ^80^ AEs are considered signs of deteriorating mental or physical health, regardless of whether the individual participates in the SPSRS‐based intervention. Participants will be encouraged to consult with the chief investigator if they notice any signs of deterioration in their mental or physical health during or after the trial. Furthermore, monitoring for serious AEs will continue after trial completion. To ensure prompt and appropriate handling of AEs occurring after trial completion, participants may contact the research team for up to one month after the final survey. This information will be conveyed to each participant during the electronic informed consent process. If an AE is reported, the research team will evaluate and document the nature, duration, severity, and outcome, as well as the participant's relationship to the study. Any AEs will be immediately reported to the Kurashiki Heisei Hospital Ethics Review Committee, which will decide whether the study should be continued, modified, or discontinued. Additionally, the research team will contact all participants and strongly recommend that they utilize relevant health services and consult mental health professionals if needed. Medical expenses related to AEs will not be covered as harm beyond that typically associated with the care provided in this trial is considered extremely unlikely.

### Trial stopping rules

The trial may be interrupted or terminated early if there are sufficient and reasonable grounds. Situations justifying termination or interruption include unexpected serious AEs as determined by the Ethics Review Committee. If an AE does occur, the research team will evaluate and record the nature, duration, severity, course, and relationship to the participant and the trial. This information will be reported immediately to the Kurashiki Heisei Hospital Ethics Review Committee, which will then decide whether to continue, modify, or terminate the study. Furthermore, even if subject recruitment is still incomplete, the trial may be terminated early based on the judgment of the Ethics Review Committee and the principal investigator.

### Research ethics approval, protocol amendments, and consent or assent

This trial has been approved by the Ethics Review Committee of Kurashiki Heisei Hospital (R07‐009). Additionally, this trial has been registered with the University Hospital Medical Information Network (UMIN) (UMIN000058947). For reasons such as research objectives or study design, should any protocol amendments be necessary, the principal investigator (with co‐investigators' consent) shall submit amendments to the Ethics Review Committee for approval. As required before implementation, electronic informed consent will be obtained from all participants, via an online questionnaire, by clicking the button indicating agreement to participate. When obtaining informed consent, clearly explaining to participants the trial's potential risks and benefits is crucial—as is ensuring they fully understand them. Therefore, before obtaining electronic informed consent, researchers will provide a written explanation of the potential risks and benefits of participating in the study, the content of the intervention (including that subliminal words such as “can” and “let us try” are displayed during the video), the assessments to be used, and the right to withdraw from participation at any time. Additionally, researchers will inform participants that participation is voluntary and that they may withdraw at any time.

### Dissemination policy

This trial will be submitted to an international peer‐reviewed journal regardless of whether the clinical hypothesis yields positive or negative results. Furthermore, we will promote wider dissemination by posting the trial overview and results on the hospital's website and by participating in and presenting at several domestic and international conferences.

## DISCUSSION

As a precursor to MDD, StD causes problems that can include decreased daily activities, reduced quality of life, worsening health status, increased economic costs, and increased mortality.^3^, ^5^, ^6^, ^7^, ^8^, ^9^, ^10^ Particularly important, however, is that StD is a risk factor for MDD.^1^, ^2^, ^6^ If effective interventions for depressive symptoms in individuals with StD are established, they could not only help maintain patients' mental well‐being but also prevent MDD's onset. Among several effective interventions for StD, SPSRS represents a groundbreaking, novel strategy: a video‐viewing website to reduce depressive symptoms.^11^, ^12^, ^16^, ^81^ The pilot RCT suggested that SPSRS can potentially improve depressive symptoms in individuals with StD.^25^ If this study replicates SPSRS's efficacy for depressive symptoms, considering its advantages (free access anywhere, ability to watch preferred videos), it could serve as an intervention for those who feel stigma about receiving psychotherapy or for individuals living in remote areas.^82^, ^83^, ^84^, ^85^ This would contribute to meeting current mental‐health needs by accommodating specific patient needs and preferences.^13^, ^14^, ^15^

As secondary outcomes in this trial, we established the K6 score for measuring psychological distress and the GAD‐7 score for measuring anxiety symptoms. Previous studies have suggested that factors influencing the transition from StD to MDD include not only depressive symptoms but also increased anxiety symptoms and psychological distress.^86^, ^87^, ^88^, ^89^ Considering that StD contributes not only to MDD but also to various other mental disorders' onset, if SPSRS also reduces psychological distress and anxiety symptoms, it could prevent not only the transition from StD to MDD but also to other mental disorders.^30^, ^90^ Despite this possibility, because the K6 and GAD‐7 scores were secondary outcomes, any observed intervention efficacy must be interpreted as preliminary. Should significant intervention efficacy be found for K6 or GAD‐7 scores, a new trial with these measures as primary outcomes would be necessary.

A previous systematic review suggested that RCTs for evaluating medical devices' efficacy (including mobile health applications) must adequately address trial design barriers, for example, randomization, participant acceptance and compliance, blinding, and determination of appropriate outcome measures.^91^, ^92^ Trials should also be carefully designed to minimize the risk of bias. Considering the Medical Research Council's methodological framework, therefore, we will evaluate SPSRS's efficacy on depressive symptoms in individuals with StD through several trials conducted in accordance with rigorous scientific methods.^93^

To minimize risk of bias, we designed this trial to address previous SPSRS studies' issues and limitations as well as their benefits. But even this trial is not without limitations. First, it was conducted exclusively with native Japanese speakers, so the results cannot be generalized across countries or cultural backgrounds. Second, the trial was conducted with individuals with no history of mental illness; therefore, its results may not apply to individuals with StD who do have a history of mental illness. Third, participants in the waiting‐list control group who wish to receive the SPSRS intervention will receive it after 5 weeks and completion of the assessment questionnaire. Although the waiting‐list control group allows us to verify SPSRS efficacy properly, we cannot entirely rule out the possibility that the waiting‐list control group's expectations or disappointment may influence the results. Fourth, this trial did not include a follow‐up assessment, so whether any efficacy persists after the intervention remains unclear. Fifth, participants will be assessed for MDD prior to the trial using the PHQ‐9 rather than a structured interview such as the MINI. Therefore, individuals with mild MDD (rather than StD) may be included as participants. Sixth, considering the burdens imposed by study participation, no follow‐up is planned after the intervention. Therefore, even if the 5‐week SPSRS intervention proves effective for depressive symptoms in individuals with StD, it remains unclear whether the therapeutic effect will persist. Previous studies have suggested that individuals with StD frequently progress to MDD 1–3 years after baseline assessment.^39^, ^94^ Therefore, future trials should include longer term follow‐up (e.g., 1–3 years) to investigate whether the therapeutic effects of SPSRS on StD symptoms are maintained and whether the intervention reduces the risk of future MDD onset. Seventh, participants for this study will be recruited via an online survey from a panel list maintained by a market research company. Therefore, the potential pool of participants will be restricted, which could introduce selection bias limiting the generalizability of the results, such as for demographic subgroups under‐represented in these panels. Eighth, we will be unable to provide mental‐health resources to candidates reporting suicidal ideation on the PHQ‐9 due to the market research company's privacy policy and terms of use. Therefore, as reported in previous studies using internet surveys, implementing best practices for addressing risks such as suicidal ideation may be difficult.^95^, ^96^ Instead, we plan to provide information on mental‐health resources upon request from participants, as simply enquiring about suicidal ideation is very unlikely to increase the risk of suicide attempt.^97^, ^98^ Nonetheless, the restriction on proactive intervention is one of the major limitations of the present trial, and future studies employing similar methodologies should seek technical solutions that incorporate real‐time safety measures without compromising anonymity. Finally, due to the intervention's nature, blinding intervention providers and participants was too difficult, so that inability might introduce performance bias, potentially skewing results in the intervention group's favor.

## CONCLUSION

This RCT investigated the efficacy of SPSRS, which presents positive word stimulation, on depressive symptoms in people with StD. If its hypotheses are supported, SPSRS is not only expected to be incorporated into routine clinical practice as an intervention strategy but may also help address contemporary mental‐health intervention issues.

## AUTHOR CONTRIBUTIONS

**Hiroyuki Uchida**: Conceptualization; methodology; project administration; writing—original draft preparation; writing—review and editing. **Takumi Igusa**: Writing—review and editing. **Chihaya Machida**: Writing—review and editing. **Tomohiro Shimada**: Writing—review and editing. **Kazuki Hirao**: Conceptualization; methodology; project administration; supervision; funding acquisition; writing—original draft preparation; writing—review and editing. All authors contributed and approved the final manuscript.

## CONFLICT OF INTEREST STATEMENT

The authors declare no conflicts of interest.

## ETHICS APPROVAL STATEMENT

This trial has been approved by the Ethics Review Committee of Kurashiki Heisei Hospital (R07‐009).

## PATIENT CONSENT STATEMENT

As required before implementation, electronic informed consent will be obtained from all participants, via an online questionnaire, by clicking the button indicating agreement to participate.

## CLINICAL TRIAL REGISTRATION

August 31, 2025, https://center6.umin.ac.jp/cgi-bin/ctr/ctr_view_reg.cgi?recptno=R000067408. This study was registered with the UMIN (UMIN000058947) in Japan.

## Data Availability

Data sharing is not applicable to this article as no datasets were generated or analyzed during the current study.
